# Added Value of Tomoelastography for Characterization of Pancreatic Neuroendocrine Tumor Aggressiveness Based on Stiffness

**DOI:** 10.3390/cancers13205185

**Published:** 2021-10-15

**Authors:** Emin Gültekin, Christoph Wetz, Jürgen Braun, Dominik Geisel, Christian Furth, Bernd Hamm, Ingolf Sack, Stephan R. Marticorena Garcia

**Affiliations:** 1 Department of Radiology, Campus Virchow Klinikum, Charité–Universitätsmedizin Berlin, Corporate Member of Freie Universität Berlin, Humboldt-Universität zu Berlin, and Berlin Institute of Health, Charitéplatz 1, 10117 Berlin, Germany; emin.gueltekin@charite.de (E.G.); dominik.geisel@charite.de (D.G.); bernd.hamm@charite.de (B.H.); 2 Department of Nuclear Medicine, Charité–Universitätsmedizin Berlin, Corporate Member of Freie Universität Berlin, Humboldt-Universität zu Berlin, and Berlin Institute of Health, Charitéplatz 1, 13353 Berlin, Germany; christoph.wetz@charite.de (C.W.); christian.furth@charite.de (C.F.); 3 Institute for Medical Informatics, Charité–Universitätsmedizin Berlin, Corporate Member of Freie Universität Berlin, Humboldt-Universität zu Berlin, and Berlin Institute of Health, Charitéplatz 1, 10117 Berlin, Germany; juergen.braun@charite.de; 4 Department of Radiology, Campus Mitte, Charité–Universitätsmedizin Berlin, Corporate Member of Freie Universität Berlin, Humboldt-Universität zu Berlin, and Berlin Institute of Health, Charitéplatz 1, 10117 Berlin, Germany; ingolf.sack@charite.de

**Keywords:** pancreatic neuroendocrine tumor, pancreas, magnetic resonance elastography, tomo-elastography, MRE, stiffness, shear wave speed, fluidity, positron emission tomography, asphericity

## Abstract

**Simple Summary:**

The prediction of pancreatic neuroendocrine tumor (PNET) aggressiveness is important for treatment planning. The aim of this study was to evaluate the diagnostic performance of magnetic resonance elastography (MRE) with tomoelastography postprocessing (tomoelastography) in differentiating PNET from healthy pancreatic tissue and to correlate PNET stiffness with aggressiveness using asphericity derived from positron emission tomography (PET) as reference. In this prospective study we showed in a group of 13 patients with PNET that tomoelastography detected PNET by increased stiffness (*p* < 0.01) with a high diagnostic performance (AUC = 0.96). PNET was positively correlated with PET derived asphericity (*r* = 0.81). Tomoelastography provides quantitative imaging markers for the detection of PNET and the prediction of greater tumor aggressiveness by increased stiffness.

**Abstract:**

Purpose: To evaluate the diagnostic performance of tomoelastography in differentiating pancreatic neuroendocrine tumors (PNETs) from healthy pancreatic tissue and to assess the prediction of tumor aggressiveness by correlating PNET stiffness with PET derived asphericity. Methods: 13 patients with PNET were prospectively compared to 13 age-/sex-matched heathy volunteers (CTR). Multifrequency MR elastography was combined with tomoelastography-postprocessing to provide high-resolution maps of shear wave speed (SWS in m/s). SWS of pancreatic neuroendocrine tumor (PNET-T) were compared with nontumorous pancreatic tissue in patients with PNET (PNET-NT) and heathy pancreatic tissue (CTR). The diagnostic performance of tomoelastography was evaluated by ROC-AUC analysis. PNET-SWS correlations were calculated with Pearson’s *r*. Results: SWS was higher in PNET-T (2.02 ± 0.61 m/s) compared to PNET-NT (1.31 ± 0.18 m/s, *p* < 0.01) and CTR (1.26 ± 0.09 m/s, *p* < 0.01). An SWS-cutoff of 1.46 m/s distinguished PNET-T from PNET-NT (AUC = 0.89; sensitivity = 0.85; specificity = 0.92) and a cutoff of 1.49 m/s differentiated pancreatic tissue of CTR from PNET-T (AUC = 0.96; sensitivity = 0.92; specificity = 1.00). The SWS of PNET-T was positively correlated with PET derived asphericity (*r* = 0.81; *p* = 0.01). Conclusions: Tomoelastography provides quantitative imaging markers for the detection of PNET and the prediction of greater tumor aggressiveness by increased stiffness.

## 1. Introduction

Pancreatic neuroendocrine tumor (PNET) is a rare but potentially deadly entity with a median overall survival of five years in patients with metastatic disease and an incidence of about 0.5 cases per 100,000 inhabitants, accounting for approximately 1–2% of all pancreatic neoplasms and 10% of all neuroendocrine tumors (NETs) [1,2]. Diagnosis is predominantly based on the clinical presentation, laboratory values (5-HIAA/chromogranin A) and imaging findings [3,4]. Nonfunctional and functional PNETs are distinguished based on the presence or absence of symptoms evoked by the excessive production of hormones such as insulin, gastrin, vasoactive intestinal peptide, glucagon, somatostatin, serotonin, growth-hormone-releasing factor and adrenocorticotropic hormone [2].

The preferred imaging modalities for the diagnostic assessment of patients with PNET are contrast-enhanced magnet resonance imaging (CE-MRI), contrast-enhanced computed tomography (CE-CT), and positron emission tomography (PET)/CT and PET/MRI with gallium-labeled somatostatin analogs like DOTATOC [4]. Multidetector computed tomography (MDCT) can contribute to discriminate between different pancreatic neoplasms [5]. PNETs often present as solid, well-circumscribed masses with contrast-enhancement in CE-CT and CE-MRI [6,7]. Somatostatin-receptor-specific imaging using PET/CT/PET/MRI provides NET-specific functional information on somatostatin-receptor expression and density with high diagnostic performance. Recently, asphericity—a measure of the spatial heterogeneity of somatostatin-receptor volume—was recognized as a valuable tool for treatment monitoring [8,9] and a predictive marker of progression-free survival [10], which is one of the primary endpoints in treatment trials [11]. Therefore, estimating tumor aggressiveness is highly desired for treatment planning. However, imaging-based estimation of aggressiveness using PET involves high radiation exposure, high costs, and is not widely available.

Tissue stiffness can be noninvasively quantified using multifrequency magnetic resonance elastography (MRE), and several studies have shown that increased stiffness can be used to detect pancreatic lesions with high sensitivity [12,13,14]. MRE with tomoelastography postprocessing (henceforth tomoelastography) improves MRE by providing higher spatial resolution and allowing for the detection of smaller structures [12,15,16]. Thus, tomoelastography is a promising imaging technique for detection and characterization of small lesions such as PNET. Tomoelastography provides another viscoelastic parameter, in addition to tissue stiffness: the loss angle of the complex shear modulus, which characterizes the internal friction of a material or its ability to move like a fluid. Since the loss angle has two bounds, 0 for pure solids and π/2 for pure liquids, it is also referred to as fluidity. Stiffness and fluidity are independent mechanical parameters that are obtained from the same MRE dataset. We hypothesized that PNET can be detected by determining the two viscoelastic parameters (stiffness and fluidity), and using quantitative thresholds as well as that tumor aggressiveness is associated with increased stiffness based on the assumption that PNET asphericity corresponds to cell proliferation.

This study is intended to provide a reference for PNET viscoelastic properties, to assess the diagnostic performance of tomoelastography in a group of participants with PNET, and to analyze PNET aggressiveness based on tomoelastography. 

## 2. Materials and Methods

This prospective study complied with the Declaration of Helsinki and was approved by the internal review board of Charité—Universitätsmedizin Berlin (EA1/076/17). Written informed consent was obtained from all participants. Tomoelastography was developed by Charité—Universitätsmedizin Berlin, and the study was performed without financial support from industry. The authors had control of the data and information submitted for publication.

### 2.1. Study Population

**Figure A1:**
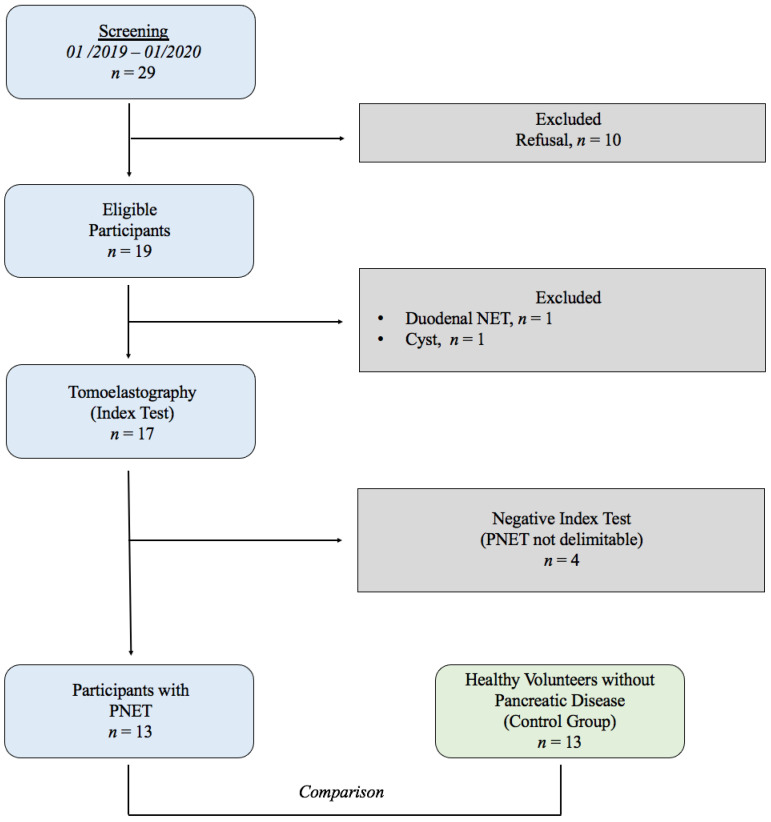
Study flowchart. PNET = pancreatic neuroendocrine tumor.

The study was conducted from January 2019 to January 2020. Nineteen consecutive participants with suspected PNET were consecutively recruited from patients presenting for routine clinical care to the department of radiology of our hospital. For inclusion, patients had to have a diagnosis of PNET based on histopathology and/or [^68^Ga]Ga-DOTATOC PET/CT/PET/MRI. Thirteen healthy age- and sex-matched subjects (CTR) without any history of cancer, pancreatic disease or alcohol abuse were included for comparison with the PNET group. Exclusion criteria for the PNET group were other final histopathological diagnoses than PNET. A flowchart of study inclusion and exclusion is provided in Figure A1.

### 2.2. Multifrequency MRE

All participants were examined in the supine position after two hours without eating and drinking. Multifrequency MRE was performed in a 1.5-Tesla MRI scanner (Siemens, Erlangen, Germany, Magnetom Aera at Campus Virchow Klinikum and Magnetom Sonata at Campus Mitte. Four drive frequencies of 30, 40, 50 and 60 Hz were induced by two compressed-air drivers placed anteriorly and posteriorly at the lower rib bone as described in the work of Marticorena Garcia et al. [12]. MRE wave images were acquired over approximately 5 minutes using a single-shot, spin-echo planar imaging (EPI) sequence with flow-compensated motion-encoding gradients [17]. Twenty-five axially oriented slices of 5 mm thickness were acquired. Protocol parameters were the same as those previously reported by Marticorena Garcia et al. [12]. For anatomical orientation, T2-weighted HASTE (half-Fourier single-shot turbo spin-echo) and T1-weighted VIBE (3D volumetric interpolated breath-hold examination) sequences were obtained.

### 2.3. Tomoelastography Postprocessing

Tomoelastography image reconstruction based on wavenumber multifrequency dual elasto-visco (*k*-MDEV) inversion was used for reconstruction of shear wave speed (SWS in m/s), which is the primary elastography parameter used for all mathematical calculations by the inversion algorithm [18]. The loss angle (φ in rad) was reconstructed by direct inversion-based MDEV. In the literature shear wave speed is considered a surrogate marker of stiffness. In this paper, we use the terms elastograms and φ-maps for quantitative SWS and loss angle maps, respectively. Both *k*-MDEV and MDEV inversion pipelines are freely available at https://bioqic-apps.charite.de (accessed on 5 August 2021). 

SWS and φ were spatially averaged and regions of interest (ROIs) were manually drawn by a radiologist experienced as an oncologic tumor board member (E.G. with >3 years of experience in clinical radiology and 2 years of elastography) supervised by (S.R.M.G. a board-certified radiologist with >8 years of experience in clinical radiology and elastography) based on elastograms and φ-maps that were matched with MRE-magnitude and T1- and T2-weighted images. In CTR participants the entire pancreas was analyzed. SWS was used as the index test, and PET/CT/PET/MRI with gallium-labeled somatostatin analogs such as DOTATOC [4] and contrast-enhanced T1-weighted MRI, acquired through clinical routine examinations, were used as imaging reference standards. On these images, PNET was identified as a [^68^Ga]Ga-DOTATOC-avid lesion and/or contrast-enhancing lesion within the pancreas [19]. In each patient with PNET, two regions were analyzed–one corresponding to tumorous tissue (PNET-T) and the other in apparently unaffected, nontumorous pancreas (PNET-NT). Necrotic areas within PNET were identified by higher T2 signal intensities and excluded from ROIs. Images were analyzed with ImageJ (Version 1.52k, Wayne Rasband, US National Institutes of Health, Bethesda, MD, USA).

### 2.4. PET Data and Evaluation of Asphericity

PET/CT/PET/MRI data were retrospectively re-evaluated (PET/CT, *n* = 7; PET/MRI, *n* = 2; median activity, 175 (range, 133–197) MBq [^68^Ga]Ga-DOTATOC; median uptake, 92 (range: 60–138) minutes) by experienced physicians specialized in nuclear medicine C.W. with >7 years of experience in nuclear medicine, supervised by C.F. with >15 years of experience in nuclear medicine). The analysis of the data was performed with a dedicated tool (ROVER, version 3.0.34, ABX advanced bio-chemical compounds GmbH, Radeberg, Germany). The metabolic tumor volume (MTV) of the primary tumor was automatically delineated in each dataset using the same threshold-based, background-adapted algorithm to determine asphericity [20]. Delineation was visually inspected and manually corrected if deemed necessary. The tumoral tracer-avidity of tissue not related to the primary tumor and delineable from the latter (such as lymph nodes and metastases) was excluded. SUV_max_ and asphericity of the MTV were calculated. The SUV was normalized using the body weight in kg. Asphericity was calculated according to the initially proposed definition [21]: S and V represent the surface area and the volume of the MTV, respectively. S was computed as the sum of all voxel surfaces forming the outer and inner surfaces of the MTV multiplied by the factor 2/3. This corresponds to the approximation of the surface area of discrete 3D objects using six voxel classes as described by [22]. It should be noted that this definition of the MTV surface area is distinct from the definition proposed by the Image Biomarker Standardization Initiative (IBSI). The IBSI estimates an MTV surface area using a mesh-based representation after the triangulation of the MTV’s outer surface [23].

### 2.5. Statistical Methods

Initially, a 2-sided power analysis was performed to determine the minimum sample size (alpha value of 0.05, beta value of 0.02, and power of 0.8). SWS and φ data showed normal distributions (unimodal, symmetric, skewness |γ| < 1). Results are given as mean ± standard deviation (SD) and 95% confidence interval (CI). Differences in SWS and φ between PNET-T vs. CTR and PNET-T vs. PNET-NT were calculated with independent and paired t-tests, respectively. Differences between metastatic and non-metastatic PNET were analyzed by unpaired t-tests. The diagnostic performance of tomoelastography in PNET was tested by receiver operating characteristics-area under the curve (ROC-AUC) analysis with 95%-CI. Appropriate cutoffs were chosen to obtain maximum specificities with acceptable sensitivity. Correlations between PNET-T SWS and PET/CT/PET/MRI maximum standardized uptake value (SUV_max_), asphericity, Ki-67, and histological grading were calculated with Pearson’s *r* and Spearman’s correlation coefficient. Statistical analysis was conducted using GraphPad Prism (v6, GraphPad Software, San Diego, CA, USA) while assuming statistically significant differences for *p* < 0.05.

## 3. Results

### 3.1. Study Population

After the refusal of ten participants to give consent during the screening period, a total of 19 MRE examinations were conducted. Two participants were excluded because their final diagnoses were a non-PNET entities (duodenal NET, *n* = 1; cystic lesion, *n* = 1) and four participants were excluded because PNET was not identifiable in elastograms (negative index test). Finally, 13 participants with PNET (mean age, 59 years; SD, 17 years; range, 24–79; 9 male) were considered for statistical analysis and compared with 13 age- and sex-matched healthy volunteers (mean age, 58 years; SD, 13 years; range—31–76; 9 male). A study flowchart is provided in Figure A1, and participant characteristics are compiled in Table 1.

### 3.2. Viscoelasticity of Pancreatic Neuroendocrine Tumor

**Figure 1:**
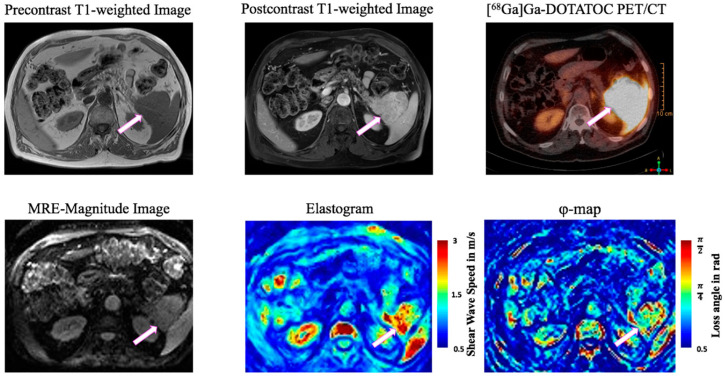
79-year-old man with pancreatic neuroendocrine tumor (PNET, white block arrow) of the pancreatic tail. Unenhanced T1-weighted image with fat saturation, postcontrast T1w image, [^68^Ga]Ga-DOTATOC positron emission tomography (PET)-CT, MRE magnitude image, elastogram and φ-map in axial plane. The tumor is characterized by low T1 signal intensity, marked contrast enhancement, PET avidity, increased stiffness (SWS) and increased loss angle of complex shear modulus (φ). Color bars represent shear wave speed (SWS) in m/s (red = high SWS, blue = low SWS) and loss angle (φ) in rad (red = high φ, blue = low φ).

**Figure 2:**
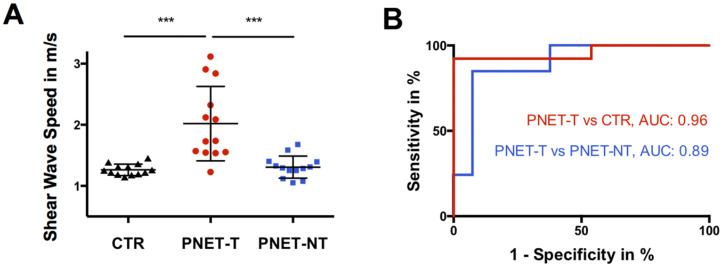
(**A**) Boxplot of shear wave speed (SWS) in m/s of controls (CTR; black triangles; *n* = 13), tumorous pancreatic region in participants with pancreatic neuroendocrine tumor (PNET-T; red circles; *n* = 13) and nontumorous pancreas in participants with pancreatic neuroendocrine tumor (PNET-NT; black squares; *n* = 13). *** *p* < 0.001. (**B**) Receiver operating characteristic curves for assessing diagnostic performance of shear wave speed in differentiation of PNET-T from CTR and PNET-T from PNET-NT with calculated area under the receiver operating characteristic curve (AUC).

Figure 1 shows a representative elastogram and φ-map along with corresponding MRI and PET/CT images of a participant with PNET. PNET-T (2.02 ± 0.61 m/s [95%-CI = 1.65–2.39 m/s]) showed higher SWS than PNET-NT (1.31 ± 0.18 m/s [95%-CI = 1.20 to 1.42 m/s], *p* < 0.001) and CTR (1.26 ± 0.09 m/s [95%-CI = 1.21 to 1.32 m/s], *p* < 0.001) (Figure 2A). An SWS cutoff of 1.46 m/s distinguished PNET-T from PNET-NT (AUC = 0.89, sensitivity = 0.85, specificity = 0.92) and a cutoff of 1.49 m/s differentiated PNET-T from CTR (AUC = 0.96, sensitivity = 0.92, specificity of 1.00) (Figure 2B). 

**Figure 3:**
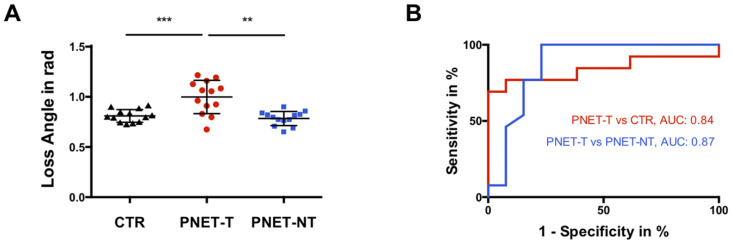
(**A**) Boxplot of loss angle of complex shear modulus of controls (CTR; black triangles; *n* = 13), tumorous pancreatic region in participants with pancreatic neuroendocrine tumor (PNET-T; red circles; *n* = 13) and nontumorous pancreas in participants with pancreatic neuroendocrine tumor (PNET-NT; black squares; *n* = 13). *** *p* < 0.001; ** *p* < 0.01. (**B**) Receiver operating characteristic curves for assessing diagnostic performance of loss angle of the complex shear modulus in differentiation of PNET-T from CTR and PNET-T from PNET-NT with calculated area under the receiver operating characteristic curve (AUC).

PNET-T (1.0 ± 0.17 rad [95%-CI = 0.90 to 1.10 rad]) showed higher φ compared to PNET-NT (0.78 ± 0.07 rad [95%-CI = 0.74 to 0.83 rad], *p* < 0.01) and CTR (0.81 ± 0.06 rad [95%-CI = 0.77 to 0.85 rad], *p* < 0.001) (Figure 3A). A φ cutoff of 0.83 rad discriminated PNET-T from PNET-NT (AUC = 0.87, sensitivity = 0.77, specificity 0.85) while a cutoff of 0.92 rad differentiated PNET-T from CTR (AUC = 0.84, sensitivity = 0.69, specificity = 1.00) (Figure 3B).

**Figure 4:**
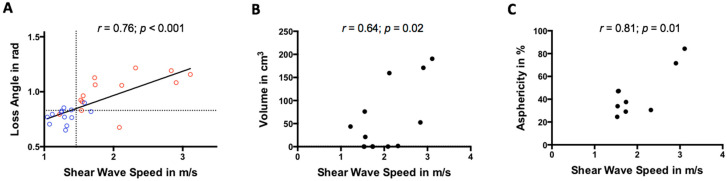
Scatter plot showing a positive correlation between shear wave speed (in m/s) and (**A**) loss angle of the complex shear modulus (cutoffs that differentiate between tumorous and nontumorous areas in participants with pancreatic neuroendocrine tumors are indicated by dashed lines; pancreatic neuroendocrine tumor = red, healthy control = blue), (**B**) tumor volume (in cm^3^), and (**C**) positron emission tomography-based asphericity (in %).

PNET-T SWS was positively correlated with φ as shown in Figure 4A (*r* = 0.76, *p* < 0.01). SWS and φ did not differ between metastatic and non-metastatic PNET (*p* = 0.31/0.93). Further details are summarized in Table 2 and Table 3.

### 3.3. Correlation with Tumor Volume, Functional Imaging and Histopathological Parameters

PNET-T SWS positively correlated with tumor volume (*r* = 0.64, *p* = 0.02; Figure 4B) while φ did not (*r* = 0.33, *p* = 0.27).

PNET-T SWS also correlated positively with asphericity (*r* = 0.81, *p* = 0.01; Figure 4C) while φ was not correlated (*r* = 0.28, *p* = 0.46). A slightly stronger correlation between PNET-T SWS and asphericity was observed in a subgroup were diagnosis was only made based on [^68^Ga]Ga-DOTATOC PET excluding those participants with missing histopathological reference (*r* = 0.94, *p* = 0.006). Neither PNET-T SWS nor φ correlated with SUV_max_ (SWS/φ, *p* = 0.9/0.35), histological tumor grade (SWS/φ, *p* = 0.51/0.30), or Ki-67 expression (SWS/φ, *p* = 0.35/0.08). Asphericity did not correlate with histological tumor grade (*p* = 0.53) or Ki-67 expression (*p* = 0.96).

## 4. Discussion

To the best of our knowledge, this is the first study to investigate elastography-derived stiffness and fluidity in participants with pancreatic neuroendocrine tumor in comparison with PET. Three important findings were made: PNETs are characterized by abnormally high tissue stiffness (SWS) and abnormally high tissue fluidity (φ), and increased stiffness is associated with greater tumor aggressiveness. 

There is increasing evidence that mechanical cues play important roles in tumor progression [24,25,26,27,28]. Fibrosis is known to account for increased tissue stiffness [29,30,31,32]. Accordingly, increased fibrogenesis in neuroendocrine tumors has been found to be promoted by various growth factors (GFs) such as transforming GF-α/β, connective tissue GF, insulin-like GF and fibroblast GF [33]. Additionally, tumor stiffness is related to altered intracellular integrins, which also serve as mechanotransducers regulating cell fate [28]. Furthermore, increases in tumor glycocalyx heterogeneity have been reported to increase cell membrane tension, resulting in altered tissue mechanics [34]. Several studies have shown that, beyond these solid structural components, vascularization and perfusion also contribute to the stiffness of biological tissues [12,15,35,36]. High tumor vascularization, which is particularly characteristic of neuroendocrine tumors [7], might thus also contribute to their greater stiffness. To the best of knowledge, only one study so far has analyzed PNET stiffness in a subgroup of seven participants using single-frequency MRE [13]. For comparison with our study, we converted the shear modulus (µ) results reported in this study to SWS using the formula SWS² = μ/ρ with ρ denoting mass density (assumed to be 1 kg/L). Consistent with our results, higher stiffness was reported for the PNET-T group with a mean (95% confidence interval) of 1.52 (1.19–2.39) m/s compared to PNET-NT with a mean of 1.09 (0.99–1.1) m/s and healthy controls with 1.1 (0.99–1.19) m/s, although mean values are slightly lower than ours [13]. Compared with pancreatic ductal adenocarcinomas, for which the group reported a mean SWS (95% confidence interval) of 2.08 (1.99–2.17) using the same tomoelastography setup, PNETs were found to be softer [12]. PNETs are morphologically and genetically different from pancreatic ductal adenocarcinomas and other pancreatic neoplasms; specifically, they are less aggressive [37] and grow more slowly [6]. The observed lower increase in stiffness we observed in PNETs might be associated with their lower fibrosis expression, which is very high in pancreatic ductal adenocarcinomas and also known as desmoplastic reaction [37]. Stiffness could therefore be an expression of tumor aggressiveness. 

Beyond this, two major characteristics reflect tumor aggressiveness: tumor volume and heterogeneity in surface molecule expression. First, larger tumors contain larger clusters of dedifferentiated cells, which is consistent with a higher tumor growth rate [38,39] and their greater ability to remodel the surrounding extracellular matrix which in turn facilitates tumor growth [40]. Second, greater heterogeneity in the expression of surface molecules such as the glycocalyx [25,34] or somatostatin-receptor proteins, which might be a further potential paradigm for tumor aggressiveness/dedifferentiation, may point to a poorer prognosis [41,42]. Tumor heterogeneity is commonly reflected by somatostatin-receptor-imaging-based parameters such as asphericity, which may therefore allow more accurate characterization of the biological behavior of NET [8,9,10]. The importance of tumor heterogeneity in [^68^Ga]Ga-DOTATATE PET or [^68^Ga]Ga-DOTATOC PET/CT was recently shown by Werner et al. [43,44], who demonstrated that heterogeneity parameters even outperformed conventional parameters like SUV, albeit they determined parameters such as tumor size variation, short zone emphasis and entropy instead of asphericity. In line with these findings, neither asphericity, nor SWS or φ correlated with SUV_max_ and total receptor expression.

Histopathology, which is based on a random biopsy and represents a small tumor portion only, does not accurately reflect intratumoral heterogeneity of Ki-67-expression, especially in intermediate G2 lesions. Unfortunately, Ki-67-expression has more pitfalls. The assessment of Ki-67 expression depends on the reporting pathologist’s expertise and is known to fluctuate in NETs, not only with the type of treatment but also over the course of therapy [45]. This might be the reason why histopathology does not correlate with stiffness, fluidity, SUV_max_, or asphericity. Similar oberservations were already noted for asphericity in gastroenteropancreatic NET prior to treatment [10]. In contrast, Weber et al. evaluated whether pretherapeutic lesion volume on ADC maps generated from [^68^Ga]Ga-DOTATATE PET/MRI potentially allows a noninvasive tumor grading. Of note, the authors observed only a weak correlation with Ki-67 for different types of NET and treatments [46]. These results should be interpreted very cautiously; the small sample size, especially, might have introduced a bias given that NETs are highly heterogeneous in terms of Ki-67 expression.

In addition to stiffness, tissue fluidity is another marker to characterize the viscoelasticity of soft tissue. Fluidity describes a material’s inherent friction, and thus its motility and deformability. Notably, fluidity is independent of water content but is an indicator of cellular adhesion in cell-rich tissues [12,16,24,47]. The disruption of adherent junctions and the perturbation of tissue polarity have been identified in tumors [28] and might explain increased friction in PNET. Similar to our observations, previous studies have reported higher fluidity in malignant entities such as pancreatic ductal adenocarcinoma [14], hepatocellular carcinoma, hepatic metastasis [16], and prostate cancer [48]. 

Although encouraging, our study had limitations. First, we investigated a small sample size without further analysis of PNET subgroups. Second, for ethical reasons, no prospective tumor biopsies were performed directly before tomoelastography. Instead, diagnoses were confirmed by routine clinical histopathology and PET/CT/PET/MRI. Third, the retrospective analysis of PET/CT/PET/MRI and histopathological data included participants with NET undergoing chemotherapy between PET/CT/PET/MRI and biopsy, which might have introduced a potential bias regarding tumor stiffness and fluidity. Further studies with prospective data analysis of both PET/CT/PET/MRI and histopathology in a larger patient population including subgroup analysis of different pancreatic tumor entities are planned for the future. 

## 5. Conclusions

In summary, tomoelastography is a noninvasive quantitative method for differentiating benign and malignant pancreatic tissue and predicting tumor aggressiveness based on stiffness. Furthermore, PNET stiffness correlates with tumor volume and PET-derived asphericity. Tomoelastography may contribute to a more reliable pretreatment risk-benefit assessment in the future. 

## Figures and Tables

**Table 1:** Characteristics of the study population (CTR and PNET).

Characteristic	CTR	PNET
Number of participants	13	13
Number of men	9	9
Number of women	4	4
Age in years		
mean ± SD	58 ± 13	59 ± 17
(range)	(31–76)	(24–79)
Body mass index in kg/m^2^		
mean ± SD	24 ± 2	27 ± 5
(range)	(20–27)	(20–35)

CTR = controls; PNET = pancreatic neuroendocrine tumor; SD = standard deviation.

**Table 2:** Parameters.

Characteristics	PNET-T	PNET-NT	CTR
SWS in m/s			
mean ± SD	2.02 ± 0.61	1.31 ± 0.18	1.26 ± 0.09
(95%-CI)	(1.65–2.39)	(1.20–1.42)	(1.21–1.32)
Fluidity expressed as loss angle in rad			
mean ± SD	1.0 ± 0.17	0.78 ± 0.07	0.81 ± 0.06
(range)	(0.90–1.10)	(0.74–0.83)	(0.77–0.85)
Entity			
Nonfunctional NET	10/13 (77%)		
Functional NET	1/13 (8%) *		
Malignant insulinoma	1/13 (8%)		
NEC	1/13 (8%)		
Tumor volume in cm^3^			
mean ± SD	55.16 ± 72.05		
(range)	(0.04–190.53)		
Tumor site (multiple locations possible)			
Head	5		
Body	7		
Tail	8		
Tumor histopathologically proven	10/13 (77%)		
Grade			
G1	1/10 (10%)		
G2	8/10 (80%)		
G3	1/10 (10%)		
Ki-67			
mean ± SD	9 ± 10%		
(range)	1–30%		
Tumor proven by [^68^Ga]Ga-DOTATOC PET/CT/PET/MRI	9/13 (69%)		
SUV_max_			
mean ± SD	45.5 ± 25.6		
(range)	(13.8–87.9)		
Time between PET/CT/PET/MRI and MRE in months			
mean ± SD	11 ± 21		
(range)	(1–68)		
Asphericity (ASP) in %			
mean ± SD	45.0 ± 20.4		
(range)	(24.5–84.3)		
Duration of disease in months			
mean ± SD	24 ± 37		
(range)	(1–128)		
Presence of metastasis	7/13 (54%)		
Drug therapy	6/13 (46%)		

* partial expression of glucagon. PNET-T = tumorous region in participants with pancreatic neuroendocrine tumor; PNET-NT = nontumorous region in participants with pancreatic neuroendocrine tumor; CTR = control; CI = confidence interval; NEC = neuroendocrine carcinoma; NET = neuroendocrine tumor; SD = standard deviation; SWS = shear wave speed; SUV_max_ = maximum standardized uptake value; PET/CT = positron emission tomography-computed tomography; PET/MRI = positron emission tomography-magnetic resonance imaging.

**Table 3:** Metrics of diagnostic performance.

	*p*	AUC	Cutoff	Sensitivity	Specificity
		95%-CI	m/s	95%-CI	95%-CI
**Shear Wave Speed**					
PNET-T vs. CTR	<0.001	0.96	1.49	92	100
		(0.88–1.04)		(64–100)	(75–100)
PNET-T vs. PNET-NT	<0.001	0.89	1.46	85	92
		(0.76–1.03)		(55–98)	(64–100)
**Loss Angle**					
PNET-T vs. CTR	0.003	0.84	0.92	69	100
		(0.67–1.01)		(38–91)	(75–100)
PNET-T vs. PNET-NT	0.001	0.87	0.83	77	85
		(0.72–1.03)		(46–95)	(55–98)

AUC = area under receiver operating characteristic curve; CI = confidence interval; CTR = control; PNET-T = tumorous region in participants with pancreatic neuroendocrine tumor; PNET-NT = nontumorous region in participants with pancreatic neuroendocrine tumor.

## Data Availability

The data presented in this study are available on request from the corresponding author. The data are not publicly available due to privacy protection.
